# Comparison of the Effectiveness of Platelet‐Rich Plasma Versus Tranexamic Acid Plus Vitamin C Mesotherapy in the Treatment of Periorbital Hyperpigmentation: A Split‐Site, Randomized Clinical Trial

**DOI:** 10.1111/jocd.16548

**Published:** 2024-09-15

**Authors:** Behzad Iranmanesh, Fatemeh Rastaghi, Najmeh Sadat Hashemi, Roxana Kaveh

**Affiliations:** ^1^ Department of Dermatology Afzalipour Hospital, Afzalipour Faculty of Medicine, Kerman University of Medical Sciences Kerman Iran

**Keywords:** periorbital hyperpigmentation, platelet‐rich plasma, tranexamic acid

## Abstract

**Background:**

Periorbital hyperpigmentation (POH) is a common cosmetic problem with a negative impact on the patient's self‐confidence, leading to a decrease in the quality of life. Current treatments include topical agents and mesotherapy, but research remains limited.

**Aims:**

Due to the undesirable effect of the available treatments, the present study was designed to compare the effectiveness of platelet‐rich plasma (PRP) injection and intradermal injection of tranexamic acid plus vitamin C mesotherapy as a therapeutic method to treat POH.

**Methods:**

Patients received an intradermal injection of PRP randomly on one side and an intradermal injection of tranexamic acid + vitamin C on the other side of their face, for three sessions with an interval of 3 weeks. Digital photography was taken, and data were assessed based on physician global assessment (PGA) and patient satisfaction.

**Results:**

Eighteen patients were studied. Among all, 12 patients had a positive family history, 2 had asthma, and 4 had a history of atopic dermatitis. Even though patient satisfaction was higher in the PRP group than in mesotherapy, it was not statistically significant. Both groups showed similar rates of improvement. However, improvement rates did not significantly differ based on various factors including gender, skin type, family history, or medical history. Age and the age of disease onset also did not significantly affect the improvement rates.

**Conclusion:**

Both methods revealed successful results in the reduction of POH. Comparing the efficacy of these two methods showed that both treatments had similar improvements.

## 1 Introduction

Periorbital hyperpigmentation (POH) is a common cosmetic problem characterized by bilateral, symmetrical brown to black‐brown macules and patches in the periorbital region [1, 2, 3].

Although this condition does not cause any significant morbidity, it has a negative impact on the patient's self‐confidence, leading to life quality decreases [1, 4]. There are a number of known etiologic factors including dermal melanin deposition, periorbital edema, post‐inflammatory hyperpigmentation secondary to atopic and allergic contact dermatitis, superficial location of the vasculature, shadowing due to skin laxity, tear trough depression and pseudoherniation of infraorbital subcutaneous fat [1, 4]. There is scarce research and data regarding the prevalence, etiology, and treatment of POH [2]. The main therapy methods consist of topical agents such as hydroquinone, kojic acid, topical retinoid acid, chemical peels, vitamin C, and sometimes a combination therapy using energy‐based devices [2, 5, 6]. Mesotherapy is a therapeutic method defined as intradermal or subcutaneous microinjection of various drugs and compounds, such as vitamin C and tranexamic acid, which provides a high concentration level of the drug in the target region [7]. Some studies have shown the efficacy of vitamin C, and tranexamic acid based‐mesotherapy in the treatment of hyperpigmentation conditions [3, 8]. Platelet‐rich plasma (PRP) is an autologous plasma with a platelet concentration two to three times higher than the normal levels. It has been proven as a safe and beneficial regenerative method, which has been widely used in aesthetic medicine [9]. It contains hundreds of bioactive agents that stimulate healing and rejuvenation [10]. A previous study showed the effectiveness of PRP injection in the treatment of hyperpigmentation conditions, such as melasma [11].

Due to the undesirable effect of the available treatments, the present study was designed to evaluate and compare the effectiveness of PRP injection and intradermal injection of tranexamic acid plus vitamin C (mesotherapy) as a therapeutic method to treat POH.

## 2 Methods and Materials

### 2.1 Study Design

Current study was designed as a split‐face, randomized clinical trial, approved by the ethics committee “code: IR.KMU.AH.REC.1399.105” and the IRTC code of “IRCT20200623047891N2”. All the ethical principles were considered during the study. All information on the study design, treatment efficacy and possible side effects was explained previously, volunteers could participate in the project after obtaining a written informed consent. Patients' private information remained confidential with the researchers.

### 2.2 Sample Size

Pilot sample was considered due to novelty of the study, estimated sample size was 22 which was confirmed by the NCSS software (NCSS, LLC). Patients were selected by simple random sampling via the “completely random” method. This involved giving each eligible participant a unique identifier and then utilizing a random number generator to select participants, ensuring there was no predetermined pattern or bias.

Individuals with POH who were referred to the Dermatology Clinic of Afzalipour Hospital, Kerman, Iran, during 2021–2023 were eligible to participate in the study.

### 2.3 Participants

Patients over 18‐year‐old with the chief complaint of POH, were considered to enroll in the study. Structural and vascular causes of periorbital darkening were ruled out through a careful physical examination and participants' history handled by a dermatologist. Other exclusion criteria included pregnancy and lactating, history of vasovagal shock during blood sampling, immunosuppression, thrombophilic or bleeding disorders, platelet count <100 000, hemoglobin below 12 in female and 14 in male, presence of any infection at the site of injection, consumption of anticoagulant agents, history of chronic diseases (chronic kidney failure, liver failure, hepatitis, cardiovascular diseases, diabetes mellitus, thyroid diseases, cancer), or history of keloid formation.

### 2.4 Intervention

In order to treat POH, patients received intradermal injection of PRP randomly on one side and intradermal injection of tranexamic acid + vitamin C on the other side of their faces for three sessions at a 3‐week interval. To prepare PRP, 10 mL of the patient's venous blood sample was taken manually with a sterile 10 mL syringe and then centrifuged with 1800 G/6 min, buffy coat and plasma were then extracted and re‐centrifuged with 2500 G/15 min. The injection of 1 mL of PRP was immediately initiated intradermally into the infraorbital area on one side of the face with a 1 cc insulin syringe 30G, (0.1 mL in each point of injection). For mesotherapy, 0.5 mL of tranexamic acid (500 mmg/5 mL Caspian Tamin pharmaceutical company) + 0.5 mL vitamin C solution (500 mg/5 Alborz Daru company) was injected intradermally on the other side of the face in the infraorbital area, using 1 mL insulin syringes, 30G, (0.1 mL in each point of injection).

### 2.5 Outcome Assessment

Patients were initially asked to fill a questionnaire of their demographic data including name, sex, age, age of disease onset, anxiety, history of alcohol consumption or smoking, history of allergic rhinitis, atopic dermatitis, and family history of POH. Patients were evaluated twice, at the beginning of the study, and 3 months following the last session of treatment. Assessments were done by two blinded dermatologists, via digital photographs, based on both physician global assessment (PGA), and patient satisfaction; (PGA score: no improvement (0%–24%), fair improvement (25%–49%), good improvement (50%–74%), excellent improvement ≥75, Patient's satisfaction assessment was according to Verbal analogue scale (VAS): 0: no satisfaction and 10: complete satisfaction).

In order to compare the side effects, patients were asked if they had any bruising, erythema, edema or necrosis on each side of their face after their previous treatment session.

### 2.6 Statistical Analysis

Data were analyzed using SPSS version 22. Normality was assessed using the Shapiro–Wilk test where an abnormal distribution of data was observed. The data were then described using frequency, median, and interquartile range. The Wilcoxon test was conducted at a significant level of 5% to compare the distribution of variables between groups.

## 3 Results

Twenty‐two patients meeting the inclusion criteria were selected for the study. Among all, four participants were excluded for the final analyses (three individuals did not complete the follow‐ups and one got pregnant during the study). A total of 18 patients, including 16 females (88.9%), and 2 males (11.1%) with the mean age of 35.55 ± 10.7 (age 17–52) years, were evaluated during the study. Most patients had a family history of POH (66.7%). A few had histories of asthma, atopic dermatitis, smoking, or alcohol consumption (Table 1). Skin types 1 and 2 were equally common (44.4%), while skin type 3 was less frequent. The mean age of disease onset was 18 years, with a standard deviation of 2.82 years, and ranged from 15 to 25 years.

**TABLE 1:** Demographic characteristics of the participants.

Asthma (%)	Atopic dermatitis (%)	Smoking (%)	Alcohol consumption (%)
2 (11.1)	4 (22.2)	2 (11.1)	1 (5.55)

### 3.1 Comparison of Patients' Satisfaction and Treatment Effectiveness Among Both Groups

In the PRP group, the average patient satisfaction score was 4.83, which was higher than that in the mesotherapy group with a score of 4.44. However, this disparity was not statistically significant (*p* = 0.58).

**FIGURE 1:**
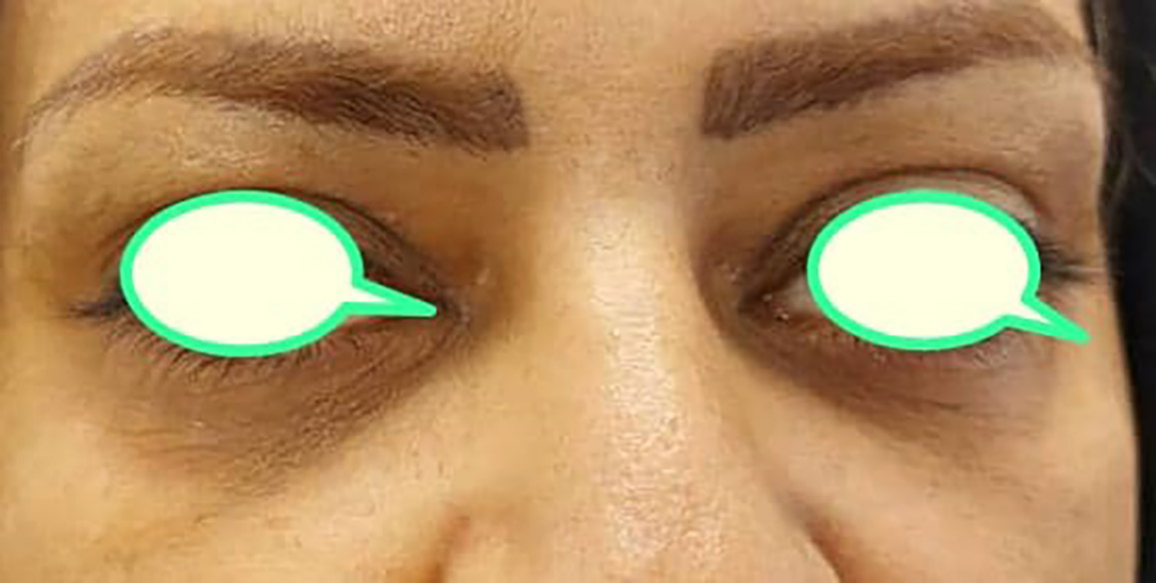
Periorbital darkness due to hyperpigmentation. Pretreatment infraorbital hyperpigmentation.

**FIGURE 2:**
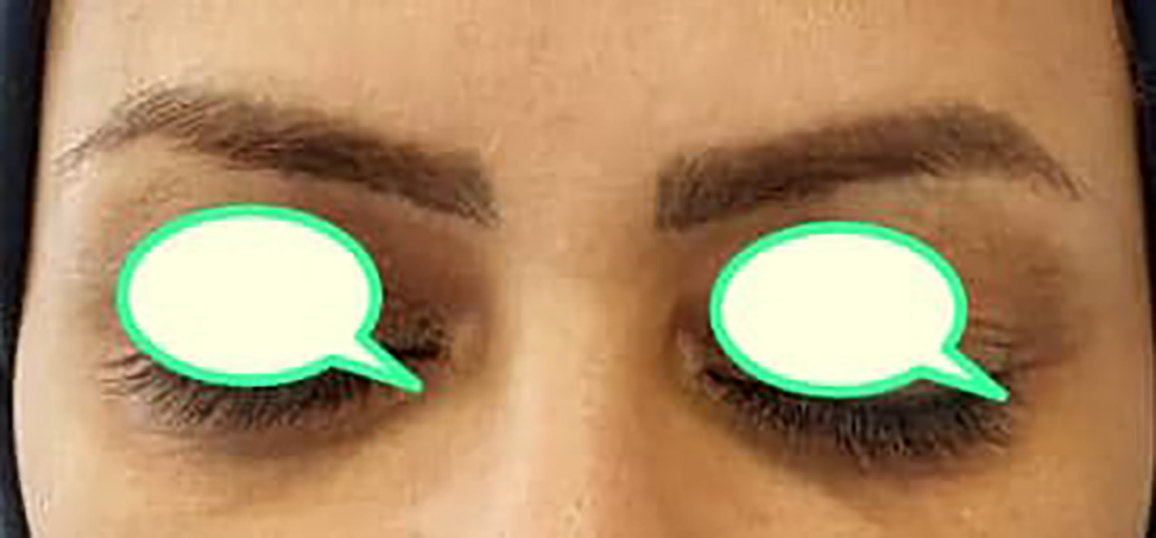
Posttreatment significant reduction in both sides' hyperpigmentation (tranexamic acid + Vit C mesotherapy was injected in the right side and PRP in the left), with equal improvement in both sides.

Both the mesotherapy and PRP groups showed the highest occurrence of improvement, within the range of moderate improvement (51%–75%). This range accounted for 33.3% in the mesotherapy group and 44.4% in the PRP group. Nonetheless, this difference was not deemed statistically significant (*p* = 0.79) (Figures 1 and 2; Table 2).

**TABLE 2:** Comparison of improvement rates between the two study groups.

	PRP frequency (%)	Mesotherapy frequency (%)	*p* value
0%–25%	2 (11.1)	3 (16.7)	0.79
26%–50%	4 (22.2)	6 (33.3)	
51%–75%	8 (44.4)	6 (33.3)	
76%–100%	4 (22.2)	3 (16.7)	

No statistically significant differences observed between the two groups in the improvement rate concerning gender, skin type, family history, asthma history, atopic dermatitis history, or smoking history (Table 3).

**TABLE 3:** Comparison of improvement rates by skin type and family history in the two study groups.

			0%–25%	26%–50%	51%–75%	76%–100%	*p* value
PRP	Skin type	1	1 (50)	2 (50)	3 (37.5)	2 (50)	0.68
		2	1 (50)	1 (25)	5 (62.5)	1 (25)	
		3	0	1 (25)	0	1 (25)	
	Family history	Positive	0	4 (100)	5 (62.5)	3 (75)	0.1
		Negative	2 (100)	0	3 (37.5)	1 (25)	
Mesotherapy	Skin type	1	2 (66.7)	3 (50)	1 (16.7)	2 (66.7)	0.42
		2	1 (33.3)	3 (50)	3 (50)	1 (33.3)	
		3	0	0	2 (33.3)	0	
	Family history	Positive	1 (33.3)	5 (83.3)	3 (50)	3 (100)	0.21
		Negative	2 (66.7)	1 (16.7)	3 (50)	0	

The frequency of improvement in the mesotherapy group based on alcohol consumption history did not have a significant difference, but in the PRP group, this difference was significant (*p* = 0.03).

Age and age of disease onset did not yield statistically significant differences in the improvement rates between the two groups.

There was no statistically significant difference between the side effects of PRP and mesotherapy groups (*p* = 0.58). None of the patients experienced severe complications.

## 4 Discussion

POH is one of the most problematic aesthetic issues, as it alters an individual's facial complexion to appear sad or tired, which can directly affect the patient's self‐confidence. It is worth noting that although some researchers have studied this subject to find an appropriate solution, only a few of them achieved desirable improvements. Therefore, the current study was conducted with the aim of finding an effective treatment for mentioned concern [3, 12]. Different articles revealed a strong association of POH with iron deficiency anemia, oral contraceptive pills (OCP) consumption, allergic dermatitis, atopy, stress, irregular menstruation, and family history of POH [1, 4, 13, 14, 15]. Our study on patients' demographic data, proposed early adulthood as the average age of disease onset, which is consistent with previous studies. Analyses of the current study revealed that most of the patients had a positive family history of POH and experienced some levels of atopia, though there were no statistically significant differences between the improvement rate of the study groups concerning gender, skin type, family history, asthma history, atopic dermatitis history, or smoking history.

A large bulk of studies has been done to find an appropriate treatment alternative for this concern. The use of PRP has been increasing in the aesthetic medicine since it contains hundreds of bioactive agents, which stimulate healing and rejuvenation. Transforming growth factor‐β (TGF β) is supposed to decrease melanogenesis, platelet‐derived growth factor (PDGF) plays some role in the synthesis of collagen and hyaluronic acid where they can improve skin tone and color [10, 16, 17, 18]. Scientists suggest an effective role for PRP in the treatment of hyperpigmentary diseases, such as melasma [11, 19, 20, 21]. Most have assessed the efficacy of PRP alone or its combination therapy with other modalities in periorbital hyperpigmentation. Some noticed a remarkable improvement in POH or the melanin index [22, 23, 24]. In a study on the comparison of the results of carbon dioxide (CO_2_) laser therapy versus CO_2_ laser + PRP, a more desirable outcome was seen in the combined therapy group [25].

Herein, we compared the efficacy of PRP and tranexamic acid + vitamin C mesotherapy in patients with POH. The results showed that most patients in both groups had moderate satisfaction. Comparing the efficacy of these methods, both groups experienced a remarkable reduction in their POH. Although, in the PRP group, a higher percentage of patients achieved good improvement, it did not make any statistically significant difference.

Studies emphasize on the importance of considering the underlying cause of POH when planning treatment. In the study of Michelle et al., it was found that PRP injection is effective due to its capacity to release growth factors and stimulate collagen production. Three trials involving a total of 60 participants demonstrated PRP's comparable efficacy to carboxytherapy. Furthermore, PRP was observed to diminish under‐eye circles and improve skin elasticity, aligning with our findings and indicating its potential for cosmetic enhancement [26]. Research by Samaan and Cartee, revealed that PRP injections significantly improved infraorbital color homogeneity, though they did not notably affect melanin content, hydration, or wrinkle volume. In contrast, weekly subcutaneous injections of carboxytherapy improved fine wrinkles and were slightly more effective in treating POH and had fewer adverse events [27].

Mehryan et al. conducted one session of PRP injection in the treatment of tear through hyperpigmentation, they showed a high improvement of infraorbital color homogeneity; however, they did not notice any significant difference in melanin content though [28]. A study by Asilian et al. revealed insignificant changes in Iranian patients with POH who were treated with three sessions of every other week of PRP, they proposed that this discordance with previous studies might be due to inadequate treatment sessions according to Iranian skin type [29]. Those three aforementioned studies found the insignificant efficacy of PRP in POH. Concordant results with the previous ones might be due to the long interval pauses between each session, inadequate treatment sessions and the small sample size of those studies. Markey et al. proposed that despite the theoretical effectiveness of PRP in the treatment of periorbital darkening, the outcomes vary. In other words, its effectiveness remains inconsistent due to diverse techniques, necessitating additional standardization and validation through clinical trials [30].

Studies on mesotherapy are associated with the intradermal or subcutaneous injection of various drugs and compounds, such as vitamin C or tranexamic acid, which provides a high concentration of the drug in the target region [7]. Some studies have shown the efficacy of vitamin C and tranexamic acid‐based mesotherapy treatment of hyperpigmentation conditions [3, 8]. Tranexamic acid reduces free arachidonic acid; thus, it diminishes tyrosinase enzyme activity, which results in a reduction of melanin synthesis in melanocytes [31, 32, 33]. Various studies have evaluated the efficacy of tranexamic acid in treating hyperpigmentary disorders. The findings suggest that tranexamic acid is an effective agent for the treatment of melasma. [8, 31, 34]. Ahmed et al. compared the efficacy of carboxytherapy, chemical peeling and vitamin c mesotherapy on POH. Although there was no significant difference between these methods, vitamin c mesotherapy showed to have had remarkable impact on improvements in pigmentation and a high level of patient satisfaction compared to other treatment options [3]. Our study confirmed previous studies regarding the efficacy of tranexamic acid in POH. To the best of our knowledge, only one trial has been performed attempting to compare the efficacy of PRP with tranexamic acid in hyperpigmentary disorders. Gharib, Mostafa and Ghonemy assessed combined therapy of microneedling + PRP versus microneedling + tranexamic acid efficacy on the melasma. They noticed that both methods could lead to the improvement in melasma, but PRP + microneedling showed greater improvements [35].

As it was revealed in the current study, intradermal injection of tranexamic acid did not have statistically significant potential in decreasing POH compared to intradermal injection of PRP.

It is worth noting that further research, involving larger sample sizes and shorter treatment intervals, is advisable to confirm the aforementioned results, given the mixed findings across various studies.

## 5 Conclusion

The present study revealed that both methods have shown successful results in the reduction of POH. Comparing the efficacy of both treatments, both treatments had similar improvement outcomes. Moreover, factors like gender, skin type, and medical history did not significantly affect results, except for alcohol consumption history, which influenced outcomes in the PRP group. These findings highlight the potential of both treatments for managing POH, though further research in larger sample groups, is needed to clarify the role influencing factors.

## Author Contributions

All authors contributed significantly to this research. They collectively designed the study protocol. Fatemeh Rastaghi, Najmeh Sadat Hashemi, and Roxana Kaveh drafted the initial paper. Behzad Iranmanesh provided critical guidance on the writing and direction. All authors revised and approved the final version. Fatemeh Rastaghi and Najmeh Sadat Hashemi assisted in the intervention under the guidance of BI.

## Ethics Statement

The current study was approved by the Ethics Committee of Kerman University of Medical Sciences (code: IR.KMU.AH.REC.1399.105).

## Conflicts of Interest

The authors declare no conflicts of interest.

## Data Availability

The data that support the findings of this study are available on request from the corresponding author. The data are not publicly available due to privacy or ethical restrictions.
